# Adverse outcome pathways as a tool for the design of testing strategies to support the safety assessment of emerging advanced materials at the nanoscale

**DOI:** 10.1186/s12989-020-00344-4

**Published:** 2020-05-25

**Authors:** Sabina Halappanavar, Sybille van den Brule, Penny Nymark, Laurent Gaté, Carole Seidel, Sarah Valentino, Vadim Zhernovkov, Pernille Høgh Danielsen, Andrea De Vizcaya, Henrik Wolff, Tobias Stöger, Andrey Boyadziev, Sarah Søs Poulsen, Jorid Birkelund Sørli, Ulla Vogel

**Affiliations:** 1 Environmental Health Science and Research Bureau, Health Canada, Ottawa, Ontario Canada; 2 Louvain centre for Toxicology and Applied Pharmacology, Institut de Recherche Expérimentale et Clinique, Université catholique de Louvain, Brussels, Belgium; 3 Institute of Environmental Medicine, Karolinska Institutet, Stockholm, Sweden; 4 Department of Toxicology, Misvik Biology, Turku, Finland; 5 Institut National de Recherche et de Sécurité, Vandoeuvre-lès-Nancy, France; 6 Systems Biology Ireland, University College Dublin, Dublin 4, Ireland; 7 National Research Centre for the Working Environment, Copenhagen Ø, Denmark; 8 Departamento de Toxicologia, CINVESTAV-IPN, Ciudad de México, Mexico; 9 Sabbatical leave at Environmental Health Science and Research Bureau, Health Canada, Ottawa, Canada; 10 Finnish Institute of Occupational Health, Helsinki, Finland; 11 Research Center for Environmental Health (GmbH), Neuherberg, Germany; 12 German Center for Lung Research (DZL), Giessen, Germany; 13 Institute of Lung Biology and Disease, Comprehensive Pneumology Center, Helmholtz Zentrum München – German, Oberschleißheim, Germany; 14 DTU Health Tech, Technical University of Denmark, Kgs. Lyngby, Denmark

**Keywords:** Nanotoxicology, Nanosafety, Novel alternative methods, Lung fibrosis, Adverse outcome pathway networks, Lung cancer, Acute inhalation toxicity, Atherosclerotic plaque formation, Lung emphysema, Occupational hazards, Toxicogenomics

## Abstract

Toxicity testing and regulation of advanced materials at the nanoscale, i.e. nanosafety, is challenged by the growing number of nanomaterials and their property variants requiring assessment for potential human health impacts. The existing animal-reliant toxicity testing tools are onerous in terms of time and resources and are less and less in line with the international effort to reduce animal experiments. Thus, there is a need for faster, cheaper, sensitive and effective animal alternatives that are supported by mechanistic evidence. More importantly, there is an urgency for developing alternative testing strategies that help justify the strategic prioritization of testing or targeting the most apparent adverse outcomes, selection of specific endpoints and assays and identifying nanomaterials of high concern. The Adverse Outcome Pathway (AOP) framework is a systematic process that uses the available mechanistic information concerning a toxicological response and describes causal or mechanistic linkages between a molecular initiating event, a series of intermediate key events and the adverse outcome. The AOP framework provides pragmatic insights to promote the development of alternative testing strategies. This review will detail a brief overview of the AOP framework and its application to nanotoxicology, tools for developing AOPs and the role of toxicogenomics, and summarize various AOPs of relevance to inhalation toxicity of nanomaterials that are currently under various stages of development. The review also presents a network of AOPs derived from connecting all AOPs, which shows that several adverse outcomes induced by nanomaterials originate from a molecular initiating event that describes the interaction of nanomaterials with lung cells and involve similar intermediate key events. Finally, using the example of an established AOP for lung fibrosis, the review will discuss various in vitro tests available for assessing lung fibrosis and how the information can be used to support a tiered testing strategy for lung fibrosis. The AOPs and AOP network enable deeper understanding of mechanisms involved in inhalation toxicity of nanomaterials and provide a strategy for the development of alternative test methods for hazard and risk assessment of nanomaterials.

## Background

Nanosafety assessment has so far relied on traditional animal-based testing. However, from the experiences of testing chemical-induced toxicity using animals, it has been realized that these conventional tests have limited predictive capacity for human health effects, are cumbersome, time & resource intensive, ethically questionable, and in most cases cost-prohibitive. More importantly, in the context of nanosafety assessment, they are not applicable as it is not feasible to generate health hazard information for hundreds of nanomaterials and their property derivatives that have found commercial application, lack toxicological knowledge and are awaiting human health risk assessment (HHRA), in a timely manner. As a consequence, cost-effective alternatives to animal testing in general such as, in vitro cell culture assays and in silico computational modeling have been earnestly desired. In fact, in vitro cell culture assays have been used as animal surrogates to test substance-induced toxicity for decades; however, the results have been mainly used to gain mechanistic knowledge of substance-induced toxicity. Owing to lack of understanding of what a majority of these in vitro alternatives measure and how the actual measurements are related to eventual adverse outcomes observed in an organism or humans, only a few of them have found regulatory acceptance for decision making. While great efforts are placed on designing and developing in vitro alternatives, the issues surrounding the sensitivity and accuracy of toxicological responses observed at the cellular level in predicting the organ or organism level effects, pose significant impediment in their uptake by the regulatory community.

A call for reduction, refinement, and replacement of animal experiments and the urgent need for animal surrogates in chemical toxicity testing was emphasized in the National Research Council’s publication on ‘Toxicity Testing for the 21^st^ Century’ [1]. The report highlighted the need for designing mechanisms-anchored in vitro assays that target the physiological pathways and key biological events perturbed following substance exposure at concentrations that trigger negative human health impact. More recently, the concept that cellular response pathways, when sufficiently perturbed by stressors result in toxicity pathways and eventually lead to adverse health outcomes, was expanded and a new concept of ‘Adverse Outcome Pathway (AOP)’ was developed, which was first applied in the ecotoxicological settings [2].

This review will briefly describe what AOPs are and their potential applications to the process of HHRA of nanomaterials. The review is structured in three parts: 1) a brief overview of the AOP concept, 2) introduction to putative AOPs that are currently explored for potential applications in nanotoxicology and 3) AOPs for design and development of alternative testing strategies in support of HHRA of nanomaterials. In the second part, the focus is placed on those AOPs, for which a roadmap exists and/or an AOP development proposal has been submitted to the Organisation for Economic Cooperation and Development (OECD) Extended Advisory Group on Molecular Screening and Toxicogenomics (EAGMST) AOP committee. Since inhalation is an important route for nanomaterial exposure and the lung is a major target organ for their toxicity, the review will describe AOPs of relevance to inhalation toxicity. Specifically, the AOPs described are for lung fibrosis, lung emphysema, acute lung toxicity, lung cancer and atherosclerotic plaque formation, adverse outcomes of specific relevance to nanomaterials. Each linear AOP is illustrated schematically and individual components of the AOP - the Molecular Initiating Event (MIE), Key Events (KEs) and the Adverse Outcome (AO) are explained. In addition, each AOP is examined for its interconnectivity with other linear AOPs and how interconnected linear AOPs will form networks representing the complexity of the biology perturbed is described. In the third part, the review will discuss how mechanistic information presented in the AOPs can be used to inform the design and development of targeted in vitro assays and for generating quality data necessary for HHRA of nanomaterials.

### A brief overview of the AOP concept

Constructing an AOP is a systematic process of collecting, organizing and describing the mechanistic information concerning a toxicological response that is initiated with the occurrence of a biological event at the molecular level (MIE) after exposure to stressors, and ensuing series of intermediate KEs that culminate in the manifestation of an AO [3]. The MIE and the AO are considered as specialized KEs. KEs describe the essential biological events at the subcellular, cellular, tissue and organ level that occur sequentially between a MIE and the AO, thus anchoring an initiating event with the eventual adverse effect. In other words, an AOP is a simplified depiction of complex toxicological processes in a linear and modular format starting with a MIE and ending with an AO. The KEs connecting the MIE and AO are selected based on their biological plausibility and measurability. The adjacent KEs are causal and describe toxicity responses at different levels of biological organization including cellular, organ, organism and population level [4, 5]. AOPs can be putative, qualitative, semi-quantitative or quantitative. Similar to the mode-of-action framework, AOPs describe the molecular mechanisms that lead to adverse outcomes; however, AOPs are substance-agnostic and thus, the description of stressors or the stressor exposure initiating the toxicity cascade is not included in the AOP [6, 7]. There are many different approaches to developing AOPs; top-down, middle-out, bottom-up, case study-based, analogy-based (extrapolation between organisms), and data-mining [8, 9]. A detailed AOP development guidance is established by the OECD [10] and a large database of AOPs describing various adverse outcomes of relevance to human and environmental health is available (https://aopkb.oecd.org/). Current efforts within nanotoxicology have particularly focused on case study-based and data mining approaches, which aim at developing new AOPs or refining existing AOPs based on one or several model stressors, which are then generalized to other stressors. The data mining approach is used when there is sufficient high-throughput and/or high-content information, such as omics data, available to identify KEs or support the development of AOPs [8, 11–13].

One of the major limitations of the linear AOPs is that they are overly simplified, reflect one single mechanism or one series of events leading to an adverse outcome and thus, may not accurately and entirely capture various events and toxicity pathways involved in the complex disease processes. It is now accepted that individual AOPs can be interconnected. Different linear AOPs that are initiated by a common MIE or converge into a single AO, or share KEs, can be interconnected in a network [14]. The interconnected AOPs form networks of AOPs. While individual linear AOPs allow simplification of the complex biology, networks of AOPs comprehensively describe the intricateness of the disease processes and hence are applicable to real world scenarios. Thus, an individual linear AOP is a building block within a larger AOP network. AOP networks are nonlinear and branched. The networks allow visualization and identification of the most upstream or downstream KEs, points of AOP convergence or divergence and also appreciation of positive and negative feedback loops [15, 16]. More importantly, the networks of AOPs will allow identification of the most commonly occurring or highly connected KEs among the AOPs, also referred to as KE nodes, which can be prioritized for testing and quantification in the absence of required experimental information on the essentiality of individual KEs [15, 16]. In the context of nanomaterials, evaluation of available linear AOPs will identify mechanisms, overlapping KEs, support prioritization of endpoints for assessment and provide clarity on property-specific influences on nanomaterial-induced toxicity.

#### Application of AOPs in HHRA of nanomaterials

Well-constructed quantitative AOPs (quantitative AOP refers to mathematical, statistical or computational models that describe complex dose, time and response-response relationships shared between the KEs and the AO [17], and the factors that modulate these relationships, enabling quantitative prediction of probability of the AO occurrence or expected magnitude of the AO at a specified exposure level [18]) can be used to gather information on chemical categories, identification and characterization of hazard, and thus, have far-reaching applications in the formal process of chemical risk assessment. In a quantitative AOP, KEs and KERs are mathematically outlined and are supported by a vast amount of quality in vivo, in vitro and/or in silico toxicity data. However, building a quantitative AOP and its evaluation is extremely onerous. It is important to note that depending on the intended applications, all AOPs need not be fully developed, quantitative or formally validated. Putative or qualitative AOPs can guide toxicity testing strategies, inform prioritization of research by identifying knowledge gaps, aid in screening and prioritization of chemicals for further toxicity testing using animal models and guide the principles of decision matrices such as integrated approaches to testing and assessment (IATA) [19–22]. AOPs can support systematic review and integration of diverse multi-source and non-standard data types, including data derived from in silico and in vitro assays that are not standard in the current risk assessment framework [23]. More importantly, putative or qualitative AOPs can inform design and development of targeted in vitro assays, identification of targeted biomarkers, providing the biological context for the interpretation and extrapolation of non-standard data to in vivo responses [6, 19, 24]. Considering the many issues related to the toxicity testing of nanomaterials, well-constructed qualitative AOPs, in the short term, will help focus research, toxicity testing and regulatory efforts, prioritize nanomaterials that require immediate testing, and aid in the development of targeted toxicity assays [25].

#### Need for nano-relevant AOPs

To date, AOP development has focused mainly on chemical-induced AOs; however, there is significant interest and efforts being made to incorporate mechanistic knowledge describing AOs of relevance to other substances, such as particles, radiation and nanomaterials [11, 12, 26]. AOPs developed for chemicals should, in general, be applicable to nanomaterials. The various biological pathways and adverse outcomes induced by nanomaterials are shown to share similarities with those induced by chemicals, albeit with a lack of detailed understanding of the MIE [12]. Research has shown that mechanistically, engineered nanomaterial-induced toxicity resembles the toxicity induced by ultrafine ambient particles [27–29] present in the natural environment. Similar to ambient particles, when deposited in the lung, nanomaterials induce oxidative stress, inflammation and cytotoxicity [30]. In other studies, some nanomaterials exhibiting properties of high aspect ratio materials, are shown to induce asbestos-like responses. Metal oxide nanoparticles are shown to induce toxicity similar to certain occupational hazards such as in miners exposed to silica, coal dust or welding fumes [31–34]. Thus, in principle, toxicity pathways and key biological events describing substance-induced AOs should be cross-applicable to chemicals and nanomaterials. However, the size-associated changes in the physico-chemical and structural properties of nanomaterials render unique material interactions with biological milieu that could enhance their toxicity potential [35]. The small size of nanomaterials compared to their bulk counterparts may result in structural defects and changes in surface groups resulting in a disrupted electron configuration, which could lead to changes in their reactivity. For example, depending on the nanomaterial chemical composition, changes in the surface properties can result in hydrophilic, hydrophobic or catalytically more or less active nanomaterials [36]. In addition to influencing the toxicity potential of nanomaterials in biological systems, their unique surface properties govern their interaction with cells that can result in cellular uptake and internalization, which is a critical biological event [37] or a MIE for nanomaterial-induced tissue responses. Many different types of nano-bio interactions have been described including physical, mechanical, chemical and receptor-mediated interactions; a single nanomaterial could initiate multiple interactions at the same time and in other cases, nanomaterials could act via non-specific interactions [12]. In addition, when present in the biological milieu, nanomaterials adsorb biomolecules such as proteins and lipids and form a ‘biocorona’ on their surface [38]. Biocorona is dynamic and its formation is influenced by the properties of nanomaterials such as surface charge, size and surface chemistry, and in return, the type of biocorona formed on the surface of nanomaterials changes their identity (physical, chemical and surface properties) [39], uptake and biodistribution. The type of biocorona also influences the host response to nanomaterials and their potential toxicity. However, because of the transitory state of a number of proteins and other biomolecules forming the biocorona, it has been difficult to identify biomolecules that influence specific aspects of nanomaterials journey. As stated earlier, while the chemical or nanomaterial-induced mechanisms of toxicity seem to follow a similar path (contrary to well-defined MIEs for chemicals such as ligand-receptor binding or protein modification), the MIEs responsible for triggering nanomaterial-induced toxicity cascades are vague (mechanical/physical damage to cellular organelles) and lack specificity [12]. To complicate it further, a nanomaterial of similar chemical composition and class exhibiting different structural properties may interact differently with the same biological microenvironment. Thus, although AOPs constructed generally for chemicals can be used to describe the AOs of relevance to nanomaterials, special considerations to domain of applicability may be needed to specify the nanomaterial property-mediated deviations in the pathway.

### AOP development strategies

While it is now accepted in the nanotoxicology community that AOPs of relevance to nanomaterials are needed and hold promise for not only identifying hazard but also for building twenty-first century toxicity assessment strategies involving animal alternatives, several issues have hampered the progress in this field, such as: 1) not all AOs induced by nanomaterials are identified as most studies to date have focused on acute responses and 2) quality data to identify KEs that would enable development of full AOPs is lacking. More importantly, how to identify KEs from the many biological events reported in the literature, especially when specific AOs are not known, is a challenge for many enthusiasts desiring to develop AOPs of interest to nanomaterials.

#### OECD Working Party for Manufactured Nanomaterials (WPMN) project on advancing the development of nano relevant adverse outcome pathways

Recently, an OECD WPMN-supported project ‘Advancing Adverse Outcome Pathway Development for Nanomaterial Risk Assessment and Categorization’ (OECD WPMN AOP project) developed, through a case study approach, a systematic methodology for identifying KEs from the existing nanotoxicology literature and demonstrated how incorporation of data on KEs can be potentially linked to AOs and lead to the development of full AOPs in the future [40]. The OECD WPMN AOP project was led by the Canadian delegation (Health Canada and Environment and Climate Change Canada) of the OECD WPMN, involved several international partners, and was supported by SmartNanoTox (Smart Tools for Gauging Nano Hazards), a European Union Horizon 2020 (H2020)-funded consortium. The project specifically focused on identifying inflammation-associated KEs since inflammation is one of the routinely assessed, observed and reported tissue response events for nanomaterials. Nanotoxicology research has established that nanomaterial-induced toxicity involves an acute inflammatory component [41–43]. The OECD WPMN AOP project reviewed a database of 191 individual studies selected from a larger collection of 11,000 studies published between 2000 and 2013, which included in vivo and in vitro reports on ~ 60 endpoints associated with inflammation for 45 different nanomaterials [40]. These individual studies were reviewed to identify KEs and the results showed that inflammation, oxidative stress and cytotoxicity events are overrepresented in the nanotoxicology literature, which are also frequently identified KEs in many of the AOPs for chemicals documented on AOPwiki (https://aopwiki.org/). Moreover, it was noted that these three KEs represent nanomaterial-induced effects at the cellular level of biological organization and share a causal relationship; persistent inflammation, oxidative stress and cytotoxicity and the consolidated interplay between the three cellular level KEs results in ‘tissue injury’, a tissue level effect or a KE. Tissue injury occurs downstream of inflammation and can be considered as a tipping point in the process of an inflammation-associated disease as it precedes tissue dysfunction [40]. Tissue injury can also be considered an AO in itself. Several AOs identified for nanomaterials such as lung fibrosis, lung emphysema, and lung cancer, have all been shown to involve these three KEs, as later discussed. Thus, the OECD WPMN AOP project marks a demonstrated step forward in the direction of providing guidance on how best to use the existing literature to populate AOP space for nanomaterials by 1) outlining a methodology to identify KEs from a vast number of reported biological endpoints in the literature and 2) establishing a database that can potentially be used to support the evaluation of existing AOPs containing the three KEs identified in the project and 3) enabling the future development of AOPs that address nanomaterial specific issues. Moreover, the established methodology and the database can be used as a good starting point for identification of novel KEs for other routes of exposures and adverse tissue effects.

#### Toxicogenomics for the development of AOPs

The other effective strategy for identifying KEs and AOs entails use of high-throughput (HT) and high-content (HC) data often referred to as toxicogenomics [9, 44]. Toxicogenomic data gives a broad overview of the molecular mechanisms of toxicity initiated by stressors in a wide variety of biological models, and as a result, is expected to feed virtually all blocks of AOPs, from the underlying toxicity mechanism to selection of an MIE, cellular level KEs, tissue and organ level KEs, and the final AO. Two main advantages of using toxicogenomics data for advancing AOP development are: i) the comprehensive data supports validation of MIEs and KEs by providing molecular level details, and ii) the data enables identification of sensitive biomarkers for targeted measurement of the KEs identified in the AOP [9, 44]. Initiatives have been taken to link biological pathway databases, such as WikiPathways, to AOPs, which enables AOP-linked bioinformatics analysis of toxicogenomics data [13, 45]. For example, a data fusion pipeline aiming to enrich AOPs with molecular detail was successfully applied to develop an AOP-linked molecular description of lung fibrosis, demonstrating that transcriptomics data captures early effects of exposure before the histological manifestation of an AO. This pipeline is readily available and applicable for development and refinement of other AOPs [13].

In another study by Nikota et al., a meta-analysis of 12 individual transcriptomics studies describing lung injury and disease responses following exposure to a variety of stressors including pathogens, chemicals, overexpression of cytokines and nanomaterials was conducted [46]. The final data included in the analysis consisted of ~ 700 individual microarray hybridizations representing 137 unique experimental conditions. The hierarchical clustering analysis of microarray data revealed robust associations between nanomaterial-induced lung transcriptomic responses with those induced by bacterial infection and chemical-induced lung pathologies. Further in depth analysis of genes found at the intersections of the clusters in the hierarchical cluster, enabled identification of specific mechanisms underlying the acute and chronic phases of nanomaterial-induced lung disease response, which was later used to establish a specific AOP for lung fibrosis [11, 47] (https://aopwiki.org/aops/173).

The computational methods described above showcase existing methodology and tools to integrate available knowledge from diverse databases and knowledge resources, to allow for data-driven learning of biological mechanisms towards development of specific AOPs [48]. Such integrated approaches enable detailed definition and description of AOP events, including support for the consideration of causality between KEs or key event relationships, which in general, are assessed via applying tailored Bradford Hill criteria, specifically refined to support AOPs [9]. Thus, the use of high-content toxicogenomics data to support tiered workflows from identifying the underlying mechanisms, defining the KEs and AO of relevance to nanomaterials to quantitative validation of AOPs is promising and has been demonstrated for chemicals [49].

A movement towards using the existing literature to support the development of AOPs of relevance to nanomaterials has been initiated and efforts are being made to create a database of nano relevant AOPs, a few of which are described below.

## Introduction to putative AOPs that are currently explored for potential applications in nanotoxicology

### AOP 173: substance interaction with lung resident cell membrane components leading to fibrosis

**Fig. 1:**
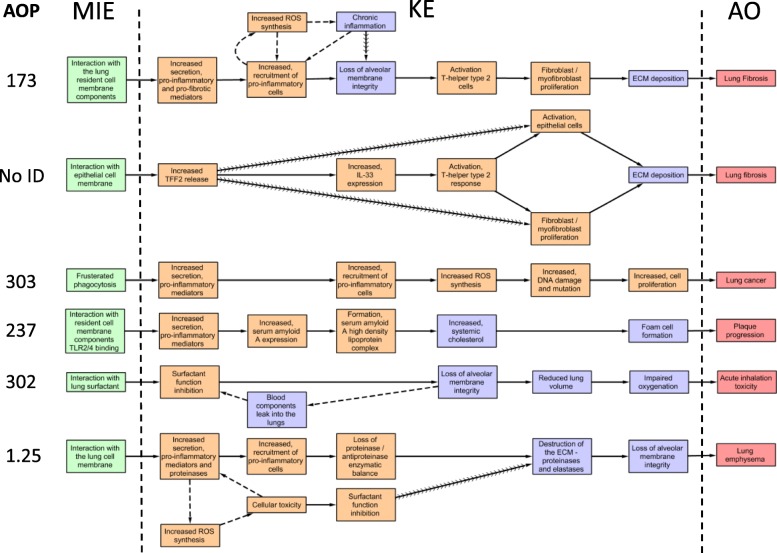
Adverse outcome pathways (AOPs) of relevance to nanomaterials. All AOPs, except one, are on the OECD EAGMST AOP work plan and are identified by the respective AOP IDs. Some AOP titles and KE descriptions may differ from how they appear on the AOPwiki. Some AOPs also include events called associative events that perpetuate the response towards adverse outcome and may be used to measure the specific KEs. Solid arrows denote adjacency (adjacent KEs occur in succession consequently to one another or immediately upstream and downstream of one another in an AOP), dashed arrows depict associative events and contiguous arrows show non-adjacency (non-adjacent KEs are further apart from each other and have other KEs in between). Green: molecular initiating events; Orange: cellular level key events; Purple: tissue level key events; Red: adverse outcomes

Lung fibrosis is an AO of the dysregulated tissue repair process. It denotes the presence of scar tissue in the localized alveolar capillary region of the lung where gas exchange occurs. It requires the presence of sustained or repeated exposure to stressor and involves intricate dynamics between several inflammatory & immune response cells, and the microenvironment of the alveolar-capillary region consisting of both immune and non-immune cells, and the lung interstitium [50]. Lung fibrosis is the most widely assessed and reported AO following exposure to nanomaterials [51]. The AOP 173 (Fig. 1) describes the qualitative linkages between interactions of substances (e.g. physical, chemical or receptor-mediated) with membrane components (e.g. receptors, lipids) of lung cells leading to fibrosis (https://aopwiki.org/aops/173). This AOP represents a pro-fibrotic mechanism that involves a strong inflammatory component and describes a mechanism that is common to both chemical and nanomaterial-induced lung fibrosis, thus demonstrating the cross-applicability of AOPs for chemical and non-chemical stressors. Briefly, in AOP 173, the MIE is described as interaction of the stressor with components of the resident lung cellular membrane, and in the context of nanomaterials, interaction with the biological microenvironment and respective players (physical, receptor-mediated, mechanical, etc.) is assumed to define the eventual adverse outcome [52]. This interaction triggers the secretion of a myriad of pro-inflammatory and pro-fibrotic mediators (KE1) that signal the recruitment of pro-inflammatory leukocytes into the lungs (KE2). KE1 and KE2 represent the same functional changes that are collectively known as inflammation. In the absence of an effective clearance of the invading stressor or in the presence of repeated stimulus, perpetuation of KE1 and KE2 or inflammation, and ensuing cell injury leads to the alveolar capillary membrane integrity loss (KE3) and activation of the T Helper type 2 (Th2) cell signaling (KE4), during which anti-inflammatory and pro-repair/fibrotic molecules are secreted. KE4 leads to fibroblast proliferation and myofibroblast differentiation (KE5), leading to synthesis and deposition of extracellular matrix or collagen (KE6). Excessive collagen deposition results in alveolar septa thickening, decrease in total lung volume and lung fibrosis (AO). As stated above, this AOP is initiated by the interaction (chemical, physical, receptor-mediated, etc) of stressors with the components of the lung cellular membrane, which, in the context of nanomaterials can be non-specific or unknown. Lung fibrosis is a well-known occupational hazard and is frequently observed in miners and welders exposed to metal dusts (https://www.thoracic.org/patients/patient-resources/breathing-in-america/resources/chapter-13-occupational-lung-diseases.pdf). It is also induced by other stressors such as, particles, pharmacological products, fibres, chemicals, microorganisms or overexpression of specific inflammatory mediators. Specific to nanomaterials, high aspect ratio carbon nanotubes (CNTs) have been shown to induce fibrosis in experimental animals (reviewed in [53, 54]). The predominant mechanism of CNT-induced fibrosis is in alignment with the mechanism outlined in AOP 173 (reviewed in [51, 55, 56]) and involves the same KEs 1–6. Although the exact mechanism for lung cell activation by CNT remains partially unclear, reactive oxygen species (ROS) are thought to activate intracellular signaling pathways and participate in inflammatory reactions. ROS can also be synthesized by pro-inflammatory cells and macrophages and in a positive feedback loop, help perpetuate the toxicity cascade towards injury and eventually the AO. The AOP 173 is specifically applicable to nanomaterials and other stressors that induce fibrosis via immune and inflammatory KEs. Additional molecular details on the intricate dynamics of the fibrotic process can also be found from the molecular description of the disease in WikiPathways [13], (https://www.wikipathways.org/index.php/Pathway:WP3624; https://aopwiki.org/aops/173). However, lung fibrosis is also suggested to be induced via non-inflammatory mechanisms. For example, direct activation and differentiation of lung fibroblasts by high aspect ratio fibers translocated to lung interstitium leading to collagen synthesis and fibrosis is reported [53, 57–64]. A network of KEs reflecting the dynamics between the fibroblasts, macrophages and epithelial cells and their role in the development of pulmonary fibrosis induced by high aspect ratio nanomaterials is summarized in a review by Vietti et al. [53]. Thus, as suggested earlier, AOP 173 reflects one of the most widely accepted mechanisms of lung fibrosis applicable mainly to a large group of pro-inflammatory stressors that are also pro-fibrotic. Lung fibrosis occurs in humans and key biological events involved are the same as the ones observed in experimental rodent models. Thus, AOP173 is applicable to a broad group of substances of diverse properties and provides a detailed mechanistic account of the process of lung fibrosis across species. AOP 173 has been fully developed and has completed external review facilitated by the OECD EAGMST AOP committee and currently under revision. The complete description of the AOP173 is publicly available (https://aopwiki.org/aops/173).

### AOP NO ID: substance interaction with lung epithelial and macrophage cell membrane leading to lung fibrosis

This putative AOP describes a deviation in the mechanism of lung fibrosis presented in AOP 173 above (Fig. 1). It specifically targets a group of stressors that exhibit asbestos-like characteristics. Asbestos and asbestos like CNTs have been shown to cause a rapid and pronounced Th2 type tissue response [65–68] that is similar to a non-canonical pathway for Th2-driven inflammation involving Trefoil factor 2 (TFF2) and interleukin 33 (IL33) [69, 70]. Thus, it describes the influence of specific material properties such as shape and high aspect ratio, in initiating the AO. Similar to AOP 173, the MIE in this AOP defines interaction between stressors and lung cells (substance interaction with lung epithelial and macrophage cell membrane). The physical interaction of asbestos-like fibres with lung epithelial cells causes cell irritation and injury, which is perpetuated due to their persistence, leading to increased release of TFF2 (KE1). Persistent increases in TFF2 levels will cause the release of IL-33 (KE2) that will generate a Th2 type response (KE3). Both the increased expression of TFF2 and Th2 response will induce epithelial cell activation and, proliferation and differentiation of fibroblasts and myofibroblasts (KE4), which in turn, leads to excessive synthesis of extracellular collagen matrix (KE5) and fibrosis (AO). Asbestos fibres cause fibrosis in humans and animal models.

### AOP 1.25 increased substance interaction with alveolar cell membrane leading to lung emphysema

Emphysema is described as the enlargement and destruction of the walls of distal and peripheral airspaces in the lung (respiratory bronchioles, alveolar ducts and alveoli) causing obstructed airflow. It is a progressive and life-threatening condition affecting alveolar structures that is irreversible once initiated. This AOP describes the interaction of stressors with alveolar cell membrane components leading to lung emphysema (Fig. 1). When the stressor interacts with the alveolar cell membrane (MIE), the resulting cell injury leads to the release of damage associated molecular patterns (DAMPs), which in turn, initiate the pro-inflammatory cascade, during which multiple pro-inflammatory mediators and proteinases are secreted (KE1) [71] that signal the recruitment of pro-inflammatory cells into the lungs (KE2). The MIE, KE1, and KE2 constitute the process of inflammation, the purpose of which is to remove the invading pathogen or toxic insult. The lingering stimulus or repeated exposure leading to cellular injury initiates repair processes, during which a variety of proteolytic enzymes are activated, and proteinases and tissue inhibitor of proteases are released by leukocytes. The proteinase-antiproteinase enzyme balance is needed to maintain lung integrity; however, the perpetuating stimulus and pro-inflammatory conditions lead to a proteinase-antiproteinase enzymatic imbalance (KE3), inducing degradation of the extracellular matrix of epithelial and endothelial cells and alveolar wall destruction (KE4) accompanied by capillary reduction in the respiratory exchange area and alveolar space enlargement (KE5). Ensuing incidences of apoptosis of alveolar cells, failed lung tissue repair, and other factors add to the lung septum damage. The inability to achieve an adequate repair balance, persistent toxicant stimuli, and increased proteolytic induction contributes to alveolar wall destruction and emphysematous lung lesions (AO). Stressors that induce emphysema include cigarette smoke [72], metal fumes or vapours, mineral dusts [73], air pollutants such as ozone and particulate matter [74], and nanoparticles [75]. This AOP is applicable to a wide variety of stressors including nanoparticles that induce an inflammatory response and affect the distal and deep lung. Inhaled nanoparticles of cadmium and lead induce alveolar emphysema in adult female mice that also show hyperemia and focal hemorrhage, with inflammatory cell infiltration and thickening of alveolar septum [76]. Exposure to aluminum nanoparticles is shown to induce emphysema-like alveolar lesions in mice [77]. A sustained lung burden of ferric oxide nanoparticles and emphysema was reported in rats post-intratracheal instillation [78]. Inhaled functionalized nanospray films with free hydroxyl groups and perfluorinate used as floor-sealing products have been shown to induce severe lung injury with emphysematous lung lesions and morphology [79]. Although in this particular study there was no evidence for the presence of nanoparticles in the product, the effects were attributed to the nanofilm that was formed after application of the product, which included inhibition of surfactant function, inititation of acute lung toxicity and development of emphysematous lung lesions. In addition, environmental nanoparticles such as carbon black derived from cigarette smoke are found accumulated in emphysematous lung tissue within dendritic cells of myeloid origin and in immune antigen presenting cells of exposed mice [75]. Emphysematous lung lesions and emphysema occurs in humans and the key events described in this AOP are mechanistically similar to the ones observed in animals, implying that the AOP presented is applicable to humans.

### AOP 237: cellular sensing of stressor leading to plaque progression

This AOP describes the linkages between substance interaction with pulmonary cell membrane components and atherosclerotic plaque progression (Fig. 1). Inhaled stressors, such as chemical substances and nanomaterials can interact with cells via physical, chemical, mechanical or receptor-mediated interactions (the MIE in this AOP) which may involve membrane lipids, surfactants, proteins and other biomolecules in the microenvironment. For nanomaterials, this interaction may be mediated by their structural attributes (such as shape) and/or surface properties (such as surface charge, surface functionality, etc). The interaction between nanomaterials and cells leads to increased secretion of pulmonary pro-inflammatory cytokines (KE1). The ensuing cytokine storm triggers the acute phase response characterized by changes in the concentration of plasma acute phase proteins. Specifically, the initial cytokine release in lungs leads to increased secretion of the lung acute phase protein serum amyloid A (SAA) (KE2) [80, 81]. Although acute phase response is conventionally assumed to be a reaction of the hepatic system, there is evidence to support the existence of acute phase machinery local to the pulmonary system and localized secretion of SAA by macrophages, fibroblasts or epithelial cells in the alveoli [82] after exposure to a variety of stressors. SAA functions as a monocyte and neutrophil chemoattractant [83]. Previous studies in mice have shown that pulmonary exposure to different types of nanomaterials induces a robust pulmonary acute phase response with increased expression of *Saa3* mRNA, which is specific to lung tissue. These studies have also shown that increases in SAA3 expression correlates with increased neutrophil influx into the lung [43, 84–92]. Furthermore, inhalation of ZnO nanoparticles induces a dose-dependent acute phase response, including increased blood levels of SAA in human volunteers [93], demonstrating that particle-induced acute phase response also occurs in humans. Increased expression of SAA leads to the formation of High Density Lipoprotein (HDL)-SAA complex (KE3). Under homeostatic conditions, HDL is in a complex with apolipoprotein A-1. The high levels of SAA displaces the apolipoprotein A-1 bound to HDL and, the newly formed HDL-SAA complex enters systemic circulation [81, 94–96]. The formation of HDL-SAA complex inhibits reverse cholesterol transport from peripheral tissues, which leads to increased systemic total cholesterol pool (KE4) [97–100] and increased foam cell formation (KE5) from macrophages in peripheral tissues [100, 101]. Foam cells are a major component of atherosclerotic fatty streaks, which reduce the elasticity of arterial walls. The foam cells promote a pro-inflammatory environment by secretion of cytokines and ROS. In addition, foam cells also induce the recruitment of smooth muscle cells to the intima, leading to arterial plaque progression (AO).

Acute phase response and the accompanying inflammatory response are strongly associated with an increased risk of atherosclerosis (as reviewed in [81]). SAA is causally implicated in atherosclerosis, since inactivation of all three inducible *Saa* genes in ApoE −/− mice lowers the formation of atherosclerotic plaques whereas overexpression of *Saa1* or *Saa3* increases atherosclerosis [102–104]. Acute phase response is an established risk factor for cardiovascular disease and blood levels of SAA and C-reactive protein (CRP) are risk factors for cardiovascular disease in prospective epidemiological studies [105]. Thus, the AOP is applicable to several types of stressors and across different species.

### AOP 303: frustrated phagocytosis leading to lung cancer

Lung cancer is one of the most prevalent cancers accounting for the highest number of deaths in the world in 2018 [106]. In general, it is a progressive disease and with a long latency period. Lung cancer is one of the adverse outcomes following exposure to fibres and particles via inhalation, which predominantly manifests in bronchial cells and rarely involves alveoli. There are two main types of lung cancer: non-small cell and small-cell lung cancer. Small-cell carcinoma represents between 10 and 15% of lung cancer [107]. Non-small cell lung cancer is subdivided in adenocarcinoma (40%), squamous cell carcinoma (25%) and large cell carcinoma (10%) [107]. Occupational exposure to fibres and particles, including high aspect ratio materials, such as asbestos, contribute to the occurrence of lung cancer [108]. It is estimated that asbestos causes 55 to 85% of occupationally related lung cancer and other diseases such as mesothelioma and results in about 233,000 deaths per year following work exposure [109]. This provides evidence that repeated inhalation exposure to, or biopersistence of, a wide variety of substances exhibiting diverse physical-chemical properties can induce lung cancer. Regardless of the substance, the underlying mechanism is the same and is initiated by the interaction of substances with lung resident immune cells (phagocytes) resulting in incomplete or “frustrated” phagocytosis (also described as failure of alveolar macrophages to phagocytize). This event serves as the MIE in this AOP.

Phagocytosis is the first line of defense against foreign invasion and therefore is essential for the maintenance of cellular and tissue homeostasis in an organism [110]. This process, mainly performed by macrophages, is divided into two steps: phagosome formation following recognition and internalization of the stressor, and phagosome maturation into a degradation compartment [110]. In lung tissue, alveolar macrophages engulf inhaled pathogens or stressors, which are then cleared from the alveolar space via the mucociliary escalator or lymphatic drainage. High aspect ratio materials (HARMs) with a ratio length/diameter ≥ 3 [111], (https://www.safenano.org/), because of their shape and rigidity, pose issues with the process of phagocytosis and lead to incomplete or frustrated phagocytosis [112]. HARMs include nanorods, nanowires, nanofibers and nanotubes among which CNTs are the most studied. Other HARMs include asbestos [113]. Some studies have investigated the effect of the length of HARMs on the capacity of macrophages to phagocytose them. The study of Sweeney et al.*,* 2015 demonstrated in primary human alveolar macrophages that treatment with long MWCNT (median length 19.3 μm) induced higher levels of MARCO receptor expression (MAcrophage Receptor with COllagenous structure, a type of pattern recognition receptor) as well as a more pronounced decreased phagocytic ability and migratory capacity than shorter MWCNT (median length 1.1 μm) [112]. Many other studies have shown that long nanotubes induce frustrated phagocytosis [114–116]. All together, these results are in good agreement with the hypothesis of Donaldson et al suggesting that fibres longer than 15 μm may cause the process of frustrated phagocytosis [117]. The consequence of incomplete phagocytosis is biopersistence of HARMs in the alveolar space [112, 114, 117, 118].

As presented in Fig. 1, frustrated phagocytosis and consequential biopersistence of substances leads to lung inflammation, characterized by increased secretion of pro-inflammatory mediators (cytokines) (KE1) and increased influx of leukocytes into lungs (KE2). In the context of biopersistent HARMs different type of cytokines, such as TNFα, IL-1β, IL-6 and IL-8 are produced, which can initiate recruitment of inflammatory cells to clear HARMs from the lung. Inflammatory cells include circulating monocytes, which differentiate into macrophages at the site of inflammation, and neutrophils. Increased cytokine secretion and modification of the metabolic patterns of the immune cells results in an increased production of ROS (KE3) [119, 120]. The ROS can act as a secondary messenger and as a mediator of inflammation. Long lasting or continuing oxidative stress leads to DNA damage and mutation (KE4) in epithelial cells [121–123]. Unlike DNA damage, DNA mutations involving both strands, cannot be repaired and are heritable. Mutations affect the genotype and potentially also the phenotype. Different mechanisms such as oxidative burst, DNA repair dysfunction or centrosome amplification and chromosome instability are implicated in DNA damage [124]. DNA damage and genetic instability could also be the consequence of direct interactions between biopersistent HARMs and chromosomes or the mitotic spindle [125]. In fact, HARMs such as asbestos and MWCNTs have been associated with DNA damage in the form of specific chromosomal aberrations, such as deletions at the tumor suppressor p16/CDKN2A site 9p21.3 (reviewed in [44]). Increased DNA damage and consequent mutations lead to an anarchical cell proliferation. Cell proliferation (KE5) is a physiological process, during which a cell replicates its genetic material and divides into two identical daughter cells. Proliferation is a highly controlled process and permit tissue homeostasis. However, when checkpoints are absent or inhibited, an increase of proliferation is observed, which is one of the hallmarks of cancer [126]. The anarchical proliferation of cells could lead to accumulation of mutations in oncogenes or tumour suppressor genes, a prerequisite for cancer development. The mechanism described in this AOP is observed following exposure to a variety of environmental toxicants in many vertebrates and is applicable to both genders regardless of the developmental stage.

### AOP 302 lung surfactant function disruption leading to acute inhalation toxicity

This putative AOP describes the linkages between the interaction of substances with the lung surfactant layer lining leading to inhibition of surfactant function and acute inhalation toxicity. Acute inhalation toxicity is the sum of all adverse effects caused following a single uninterrupted exposure to a substance via inhalation over a short period of time (24 h or less) [127]. Acute lung toxicity in humans is characterized by cough, difficulty in breathing, tightness in the chest, fever and vomiting (AO) [128, 129].

Respirable substances that are small enough to evade normal respiratory surveillance and reach deeper lung regions, come in direct contact with lung surfactant, which provides the first defense barrier. This interaction between the substances and the surfactant marks the MIE in the AOP for acute inhalation toxicity (Fig. 1). The interaction with surfactant results in disruption of lung surfactant function (KE1). The main function of lung surfactant is to lower the surface tension at the air-liquid interface during the breathing cycle. The substance interaction mediated surfactant function disruption leads to an increase in minimum surface tension and alveolar collapse. Collapsed alveoli, if reopened, exert shear stress on the epithelium or if remained closed, lead to reduced lung volume, decreased area for gas exchange and reduced blood oxygenation. Regardless, collapsed alveoli lead to loss of alveolar capillary membrane integrity (KE2), resulting in bleeding into the lungs (KE3). Bleeding and consequent release of blood components such as albumin and fibrin into the air-liquid interface will further disrupt lung surfactant function (KE1) [130–133] leading to exacerbation of the process. The collapse of the alveoli results in reduced lung volume (KE4) and hypoxia. The combination of bleeding into the lungs and impaired blood oxygenation leads to acute inhalation toxicity (AO).

When nanomaterials reach the alveoli and come into contact with the lung surfactant, they are immediately covered by a lung surfactant corona. This corona is distinct from the corona that forms if nanomaterials come into contact with serum, as it consists mainly of phospholipids rather than proteins [134]. Both phospholipids and surfactant associated proteins are essential for the function of lung surfactant and their binding by nanomaterials can deplete both the phospholipids and associated proteins from the air-liquid interface, thus disrupting function. The physicochemical characteristics of the surface of the nanomaterials determine what binds to the surface, i.e. hydrophobic nanomaterials bind relatively more to phospholipids than hydrophilic nanomaterials [134]. A wide range of nanomaterials have been shown to affect the function of lung surfactant in vitro including metal and carbon-based nanomaterials [135–143].

Acute inhalation toxicity is observed frequently in humans [128] and in experimental animals [129]. Lung surfactant function impairment in vitro has been strongly associated with induction of acute inhalation toxicity and acute lung injury both in humans and in experimental animals [128].

#### Network of AOPs

**Fig. 2:**
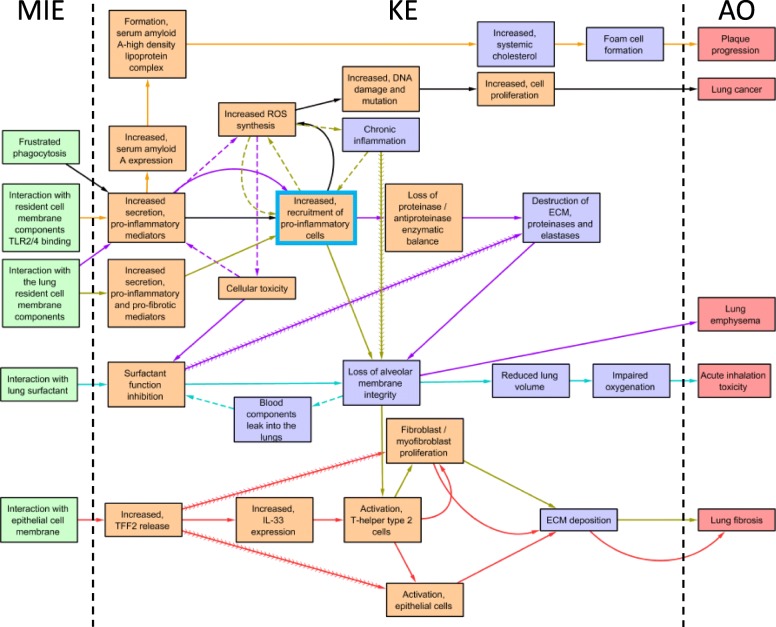
Network of adverse outcome pathways (AOPs). Individual linear AOPs were combined into a derived network based on commonly shared KEs and KERs. The network was then visualized and interpreted using a software called *Cytoscape 3.7.2* (https://cytoscape.org/), an open-source platform for construction, analysis and visualization of biological networks [145]. In *Cytoscape*, KEs were represented as nodes (or vertices) in a network and KERs as edges connecting nodes. The network was directionally analyzed (meaning the directional relationship between source and target nodes is preserved) using the built-in plugin *NetworkAnalyzer 2.7*, which computes the topological parameters of the network (such as edge count, and centrality measures) in a directional, or non-directional manner . Six AOPs were used to build the derived AOP network for inhalation toxicity induced by nanomaterials. For visualization purposes, the KERs (depicted as arrowed edges) of each AOP were color coded to clearly highlight which pathway they are associated with. KE node positions (i.e. final placement of the KE node in the graphic, but not connectivity) were manually set to maximize readability and space. This does not modify the results of the topological network analysis. Adjacent KERs are depicted as solid arrows. Associative KERs are highlighted as dashed arrows in the network, but were otherwise treated normally for the directed network analysis. Non-adjacent KEs are depicted using contiguous arrows. The most hyperconnected KEs were determined to be the nodes with a definite in-degree/out-degree ratio > 0 (indicating there are inputs and outputs from the node making it a KE in an AOP) and the highest edge count (number of connecting KERs) following network analysis. Convergent KEs were defined as having a larger in degree than out degree parameter (more relationships feed in than leave this KE). Similarly, divergent KEs were defined as having smaller in degree than out degree values (More relationships branch out from this KE than feed into it). Green: molecular initiating events; Orange: cellular level key events; Purple: tissue level key events; red: adverse outcomes. Arrows are colored based on corresponding AOP. Solid arrows represent adjacent key events. Dashed arrows represent associative events. Contiguous arrows represent non-adjacent KEs. The most highly connected KE is bordered in blue

Individual linear AOPs presented in Fig. 1 were used to build a network of AOPs for inhalation toxicity induced by nanomaterials using *Cytoscape* [144] (Fig. 2). A directed network analysis, using the built-in plugin *NetworkAnalyzer* in *Cytoscape,* was carried out in order to determine topological characteristics of the derived network (Table 1). From the derived network, it was observed that each AOP shares at least one common KE. The MIE defined as ‘interaction with lung cell membrane component’ is common to most AOPs in the network differing only in specific details such as cell types or biomolecules involved in the interaction. For example, in AOP 173, the interaction between the stressor and lung cells is not specific, whereas, in the other AOPs, the specific details are described such as specific cell types or the biomolecules involved. In AOP 303 for lung cancer, the MIE is defined as frustrated phagocytosis, which is also described as a type of interaction that triggers the biological cascade leading to lung fibrosis in AOP 173. As stated earlier, MIEs in the nanomaterial relevant AOPs are non-specific and/or may involve multiple interactions at the same time. A select set of HARMs such as CNTs are shown to induce physical or mechanical interaction involving frustrated phagocytosis and, at the same time, can bind to receptors such as toll-like receptors or selectins [43], initiating the KEs of inflammation and immune responses at the cellular level. Among the KEs, the hub KEs of inflammation, including altered expression of pro-inflammatory mediators and increased recruitment of leukocytes, are common across the AOPs in the network. However, the description of the KE ‘altered expression of pro-inflammatory mediators’ was represented with certain variations to include specific players such as cell types or objects to highlight the tissue or AO specific details. For example, in the AOP 173, this KE was described as altered pro-inflammatory and pro-fibrotic mediators, and in the AOP 1.25  for lung emphysema, it was defined as altered expression of pro-inflammatory mediators and metalloproteinase. There was also a high degree of overlap between the KEs describing loss of alveolar capillary membrane integrity (common to three AOPs and three AOs), and activation of Th2 type response, fibroblast/myofibroblast proliferation and extracellular matrix deposition (two AOPs, one  AO). In addition, the associative events (a biological event that helps propagate the response but may not be essential to be listed as a KE in the pathway) or cyclic (the positive feedback loops) KERs describing increased oxidative stress and cytotoxicity were common to two AOPs. Oxidative stress was defined as an associative event in two AOPs and was a KE in one of the AOPs. The most highly connected KE (‘Increased, recruitment of pro-inflammatory cells’) is related to inflammation, which represents a tissue level KE. The most central KE was ‘Loss of alveolar membrane integrity’, a tissue level KE reflecting tissue injury, which was identified as one of the critical events potentially preceding nanomaterial-induced tissue dysfunction and adverse outcome [40]. The acute cell injury, oxidative stress, and inflammation triggered immediately following exposure to nanomaterials act in a positive feedback loop mechanism propagating the initial inflammatory response, resulting in tissue injury. This is a potentially decisive state leading to adverse outcomes of lung fibrosis, lung emphysema and acute inhalation toxicity. From the network, it is clear that nanomaterial-induced pathologies have a similar origin and the eventual adverse outcome trajectory may be influenced by the property variations, duration, and time of exposure which suggests certain nanomaterials may have the capacity to induce multiple AOs. Among the AOPs presented in this network, only AOP 173 has been fully developed and is considered qualitative at present. Regardless, the putative AOPs in the network can be used to inform the focus of interim research activities, focusing on overlapping KEs for targeted analysis by specific endpoints.

**Table 1:** Directed network analysis for the derived AOP network built in Cytoscape and analyzed using the NetworkAnalyzer plugin

Node	MIE / KE / AO	# Shared AOPs	Betweenness Centrality	Edge Count	In degree	Out degree	In/Out Ratio
Increased, recruitment of pro-inflammatory cells	KE	3	0.088235	9	5	4	1.25
Loss of alveolar membrane integrity	KE	3	0.127451	8	4	4	1
Increased secretion, pro-inflammatory mediators	KE	3	0.0918	8	4	4	1
Increased ROS synthesis	KE	3	0.054367	7	3	4	0.75
Activation, T-helper type 2 cells	KE	2	0.057041	5	2	3	0.666666667
Surfactant function inhibition	KE	2	0.032531	5	3	2	1.5
ECM deposition	KE	2	0.018717	5	3	2	1.5
Fibroblast / myofibroblast proliferation	KE	2	0.016934	5	3	2	1.5
Increased, TFF2 release	KE	1	0.005348	4	1	3	0.333333333
Cellular toxicity	KE	1	0.027184	3	1	2	0.5
Activation, epithelial cells	KE	1	0.016934	3	2	1	2
Destruction of ECM, proteinases and elastases	KE	1	0.010695	3	2	1	2
Chronic inflammation	KE	1	0.004902	3	1	2	0.5
Increased, serum amyloid A expression	KE	1	0.032086	2	1	1	1
Formation, serum amyloid A-high density lipoprotein complex	KE	1	0.026738	2	1	1	1
Reduced lung volume	KE	1	0.026738	2	1	1	1
Increased, systemic cholesterol	KE	1	0.019608	2	1	1	1
Increased, DNA damage and mutation	KE	1	0.016043	2	1	1	1
Impaired oxygenation	KE	1	0.01426	2	1	1	1
Foam cell formation	KE	1	0.010695	2	1	1	1
Increased, cell proliferation	KE	1	0.008913	2	1	1	1
Loss of proteinase / antiproteinase enzymatic balance	KE	1	0.006684	2	1	1	1
Increased secretion, pro-inflammatory and pro-fibrotic mediators	KE	1	0.006239	2	1	1	1
Blood components leak into the lungs	KE	1	0.005348	2	1	1	1
Increased, IL-33 expression	KE	1	0.001783	2	1	1	1
Lung fibrosis	AO	2	0	2	2	0	Undefined
Interaction with the lung resident cell membrane components	MIE	2	0	2	0	2	0
Lung cancer	AO	1	0	1	1	0	Undefined
Plaque progression	AO	1	0	1	1	0	Undefined
Acute inhalation toxicity	AO	1	0	1	1	0	Undefined
Lung emphysema	AO	1	0	1	1	0	Undefined
Frustrated phagocytosis	MIE	1	0	1	0	1	0
Interaction with resident cell membrane components TLR2/4 binding	MIE	1	0	1	0	1	0
Interaction with epithelial cell membrane	MIE	1	0	1	0	1	0
Interaction with lung surfactant	MIE	1	0	1	0	1	0

Betweenness centrality reflects the amount of control a KE exerts on other KEs in the network, a higher number indicates greater degree of control (and greater network disruption if removed)

*MIE* molecular initiating event, *KE* key event, *AO* adverse outcome

### AOPs for design and development of alternative testing strategies in support of HHRA of nanomaterials

As stated earlier, one of the major hurdles that has impeded nanomaterial safety assessment is lack of quality data. Owing to their number and many forms, complete assessment of just the first generation nanomaterials via conventional animal testing was estimated to require approximately 50 years or more and billions of dollars in funding. With the appearance of next generation advanced materials in the market, this task is projected to be even more onerous. The in vitro and in silico alternatives to animal testing are being actively developed; however, the incorporation of an AOP framework in the design of such alternatives, would enable effective interpretation of data generated and their extrapolation to responses potentially observed in animals and humans. One of the successful examples supporting the use of AOPs in decision making process is the story of skin sensitization (AOP 40), the first of the AOPs to be endorsed by the OECD. Since its endorsement, in vitro tests developed for multiple KEs identified in this AOP have found regulatory acceptance, OECD test guidelines have been established [146] and various test guidelines for in vitro skin sensitization assays are available (https://www.oecd-ilibrary.org/environment/oecd-guidelines-for-the-testing-of-chemicals-section-4-health-effects_20745788). The efforts are well underway in support of recommendations to completely replace the animal-based skin sensitization tests by AOP-informed suite of in vitro, in silico and in chemico tests. Thus, well-constructed AOPs will allow effective use of mechanistic knowledge in the design and development of animal alternatives, enabling generation of quality toxicology data that incorporate dose-response relationships and cross-species relevance, resulting in quantitative risk assessments (http://www.oecd.org/chemicalsafety/testing/series-testing-assessment-publications-number.htm).

**Fig. 3:**
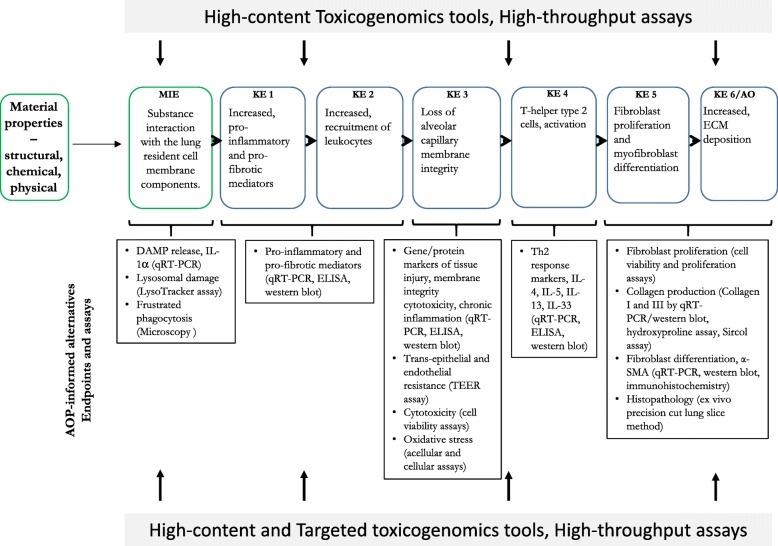
Schematic representation of AOP 173: Substance interaction with the lung resident cell membrane components leading to lung fibrosis https://aopwiki.org/aops/173. Individual example assays and endpoints assigned to specific KEs that can be used in AOP-informed alternative testing strategies. The list of assays is not exhaustive. Of note, the development of the AOP, as well as identification of targeted endpoints for KE assessment resulting in validation of the AOP modules can be supported by HT and HC methods, including whole genome (hypothesis generating) or targeted (predictive testing) toxicogenomics. The exposure models can vary and may include submerged mono-cultures or advanced models (Air Liquid Interface, co-cultures, 3D models, lung slices, etc)

**Fig. 4:**
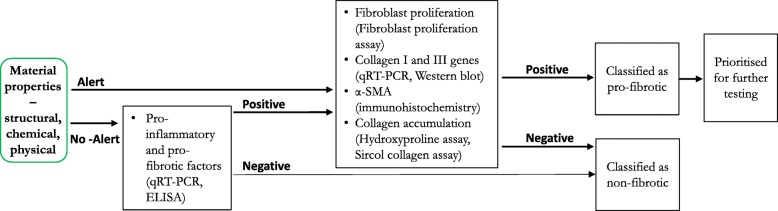
A simple AOP-informed tiered testing strategy for nanomaterial-induced lung fibrosis. Alerts refer to physical-chemical or structural features of nanomaterials

The suitability of an AOP for applications in research or regulatory decision making is solely based on the biological accuracy of the mechanism presented, biological plausibility and measurability of KEs and the weight of evidence presented in support of KERs. AOP 173 for lung fibrosis has undergone internal and external review by the OECD EAGMST AOP committee and the external subject matter experts, respectively and is currently under revision. In Fig. 3, the well accepted mechanism of lung fibrosis (AOP 173) is presented with a number of non-animal assays that can potentially be used to assess different KEs. Table 2 lists the KEs in AOP 173 (identified by their ID on AOPwiki) and specific cell types, endpoints and assays used for measuring the specified KEs in nanotoxicology literature. This information can be used to build a non-animal testing strategy for predicting nanomaterial-induced lung fibrosis. To be effective, the strategy must include a combination of assays and endpoints targeting more than one KE in AOP 173. While a number of assays have been identified, what is not clear for now is which assays or endpoints should be prioritized and the rationale for prioritization of one assay or endpoint over the other is lacking. For example, in the case of inflammation, the list of specific actors and cell types used to measure higher order KEs of inflammation depends on individual researcher’s expertise, resources and experiences. The heterogeneity in assay types, objects assessed or cell type used poses significant challenges in interpreting the data derived from these assays. Moreover, questions such as 1) do all biomarkers bear equal sensitivity in predicting in vivo inflammation, 2) how many pro-inflammatory mediators should be assessed at a minimum and 3) how is altered expression defined and if the definition can be applied across different biomarkers of inflammation consistently, have to be addressed. A criterion for selection of biomarkers from the vast number of pleotropic and redundant pro-inflammatory mediators should be carefully established and guidance regarding the minimum set of cell-type-specific object changes that represent occurrence of a given KE needs to be defined. In order for such prioritization or for developing animal alternative testing strategies for lung fibrosis, a common dataset generated using the different assays and endpoints listed in Fig. 3 or Table 2, for different nanomaterials and their variants is required. The resulting outcome of such an analysis will include thousands of data points from a heterogenous group of assays and endpoints describing dose-responses for several nanomaterials and their property variants. The integrated analysis of all data and interpretation of such data will require sophisticated and dynamic computational modelling approaches. Some nanoinformatics infrastructure for such an undertaking already exists. Models developed as part of ToxCast and Tox21 initiatives for chemicals can be used, which will enable integration of data from multiple assays and aid in the interpretation of data describing the biological relevance and identification of point-of-no return or tipping points associated with dose-dependent transitions from adaptive cellular responses to adverse outcomes [147]. It is important to note that in all of the AOPs involving early inflammatory and immune events, initiation and progression of early inflammatory KEs towards the AO greatly depends on the relative balance of damage & repair, stressor characteristics, time, and exposure conditions, all of which may limit the predictive potential of an in vitro strategy. Thus, a well-thought combination of KEs and advanced in vitro models for measuring them may be required to attain effective predictions of outcomes. Certain properties of nanomaterials such as shape, aspect ratio, chemical composition can serve as alerts or triggers for an MIE; however, property alerts for all nanomaterials have not been identified, necessitating testing of most suspected nanomaterials using multiple assays and endpoints. Until a validated strategy is available, a simple decision tree as shown in Fig. 4 can be used in a tiered testing strategy involving Tier-1 measurement of higher degree inflammatory KEs and Tier-2 testing of KEs downstream of inflammation representing histological manifestation of the disease. Since standardized protocols and guidance on testing are not available, each KE may have to be assessed using more than one assay and multiple endpoints. As the data generated from using these assays increases, the strategy can be improved by selecting most predictive parameters or by including additional parameters to define predictive efficiency. In addition, as more data becomes available, criteria for evaluating the in vitro methods addressing aspects such as, what KE is assessed or predicted by the assay, how close is the measured endpoint to the response observed in vivo, how many nanomaterials have been tested using the method, does the method allow dose-response analysis and is there a standard operating protocol available, can be developed to select the assays and endpoints that demonstrate the best predictive potential. In a recent study by Rahman et al.*,* (2020), a testing strategy involving a combination of a transcriptomic signature consisting of 17 genes (referred to as PFS17) predictive of lung fibrosis targeting different KEs in the AOP 173 and an ex vivo precision cut lung slice method was proposed as a promising alternative to assess lung inflammation and lung fibrosis induced by nanomaterials [148]. Although these strategies and specific methodologies are well described for chemicals [147], they are not readily applicable for nanomaterials due to lack of data. The novel approaches described here are still in their infancy in the context of nanomaterials and will require further validation before their routine integration into nanomaterial research and regulation.

**Table 2:** Key events (KE) contained in AOP 173 (https://aopwiki.org/aops/173). Markers, cell types and assays were identified in Vietti et al.*,* 2016 and Nymark et al.*,* 2018. WP numbers refer to Wikipathways (https://www.wikipathways.org/index.php/WikiPathways). Relevance of the biomarkers for predicting lung fibrosis is described as A (association between in vitro and in vivo data for the biomarker), B (biomarker involved in the AO as demonstrated with deficient or transgenic mice, inhibitors, etc), C (biomarker strongly associated with the AO), D (biomarker identified by data mining). ELISA: enzyme-linked immunosorbent assays; EPR: electron paramagnetic resonance; GO: Gene Ontologies; qRT-PCR: quantitative reverse transcription-polymerase reaction; WB: western blot

KE #	KE	Biomarkers	Cell type	Assay	Relevance
1495	Interaction with the resident cell membrane components	Toll-like receptor signaling WP75 (CXCL8, CCL3, CCL4, CCL5)	Epithelial cells	Transcriptomics or individual assays (qRT-PCR)	D
		DAMPS/alarmins (IL-1a)	Macrophages	ELISA, qRT-PCR	C
1496	Secretion of proinflammatory and profibrotic mediators	ROS	Macrophages, fibroblasts	EPR (acellular), HO-1 (cellular, ELISA, RT-PCR)	C
		p38 MAPK	Fibroblasts	WB	C
		NF-KB	Macrophages	WB	C
		MAP kinase	Epithelial cells	WB	C
		IL-1b (+ NADPH oxidase and inflammasome)	Macrophages, epithelial cells	ELISA, WB (± NADPH oxidase or inflammasome inhibitors)	A, B, C
		TNF-a	Macrophages	ELISA, WB (qRT-PCR)	C
		IL-18	Epithelial cells	ELISA, WB (qRT-PCR)	C
		IL-8	Epithelial cells	ELISA, WB (qRT-PCR)	C
		TGF-b	Macrophages, fibroblasts, epithelial cells	ELISA, WB (qRT-PCR)	B, C
		PDGF	Macrophages, fibroblasts, epithelial cells	ELISA, WB (qRT-PCR)	C
		Cytokine and inflammatory response WP530 (PDGFA, CXCL2, CSF3, CSF2, IL12B, IL13, IL4, IL5, IL6)	Epithelial cells	Transcriptomics	D
		Chemokine signaling WP3929 (CCL2, CCL11, CCR2, CCR3)	Epithelial cells	Transcriptomics	D
1497	Recruitment of inflammatory cells				
1498	Loss of alveolar capillary membrane integrity	Transepithelial/transendothelial electrical resistance (TEER)	Endothelial cells, epithelial cells	Ohmic resistance or impedance	C
		ROS	Macrophages, fibroblasts	EPR (acellular), HO-1 (cellular, ELISA, qRT-PCR)	C
1499	Activation of T (T) helper (h) type 2 cells	Chondrocyte differentiation WP474 (CTGF, TGFA, GREM1, ATP11A)	Epithelial cells	Transcriptomics	D
		Matrix metalloproteinases WP129 (MMP9, MMP2, TIMP1)	Epithelial cells	Transcriptomics	D
		TGFB signaling WP560 (SKIL, SPP1)	Epithelial cells	Transcriptomics	D
		Differentiation pathway WP2848 (EFG, IGF1, HGF, FGF1, FGF2, FGF7)	Epithelial cells	Transcriptomics	D
		Cytokine and inflammatory response WP530 (PDGFA, CXCL2, CSF3, CSF2, IL12B, IL13, IL4, IL5, IL6)	Epithelial cells	Transcriptomics	D
		Chemokine signaling WP3929 (CCL2, CCL11, CCR2, CCR3)	Epithelial cells	Transcriptomics	D
		Leukocyte/Myeloid cell differentiation GO: 0045637/GO: 1902105 (CALCA, CEBPB)	Epithelial cells	Transcriptomics	D
		TGF-b	Macrophages, fibroblasts, epithelial cells	ELISA, WB (qRT-PCR)	B, C, D
		PDGF	Macrophages, fibroblasts, epithelial cells	ELISA, WB (qRT-PCR)	C, D
1500	Fibroblast proliferation and myofibroblast differentiation	Smad	Fibroblasts, epithelial cells	WB	C
		ERK1/2	Fibroblasts	WB	A
		fibroblast proliferation	Fibroblasts	cell count, cell viability assays	A
		fibroblast differentiation (a-SMA)	Fibroblasts	qRT-PCR, WB	C
		epithelial-mesenchymal transition, EMT (ZO-1, SP-C, E-Cad, fibronectin, FSP-1, a-SMA, vimentin)	Epithelial cells	qRT-PCR, WB	C
		MAPK signaling WP382	Epithelial cells	Transcriptomics	D
		p38 MAPK WP400	Epithelial cells	Transcriptomics	D
		TGFB signaling WP560 (SKIL, SPP1)	Epithelial cells	Transcriptomics	D
		TGF-b	Macrophages, fibroblasts, epithelial cells	ELISA, WB (qRT-PCR)	B, C, D
		PDGF	Macrophages, fibroblasts, epithelial cells	ELISA, WB (qRT-PCR)	C, D
		Chondrocyte differentiation WP474 (CTGF, TGFA, GREM1, ATP11A)	Epithelial cells	Transcriptomics	D
		Differentiation pathway WP2848 (EFG, IGF1, HGF, FGF1, FGF2, FGF7)	Epithelial cells	Transcriptomics	D
1501	Extracellular matrix deposition	Collagen production (Collagen I and III or soluble collagen)	Fibroblasts	qRT-PCR, WB, Sircol assay	A
1458	Pulmonary fibrosis

#### Cell type, exposure model and biomarker selection

In addition to identifying relevant assays for consideration in testing, AOPs also provide a rationale for selecting biomarkers for inclusion in targeted analysis and relevant cell types for specific assays. However, AOPs do not help identify the most relevant in vitro exposure model. Simple cell culture models such as submerged mono-cultures involving lung cells have been routinely used in in vitro testing; however, with limited sensitivity to predict in vivo lung activity of nanomaterials. Efforts are currently underway to design and optimize advanced lung models consisting of physiologically relevant aspects of lung exposure and response. For example, exposure models that mimic the air-liquid interface at the epithelial surface and co-cultures of lung cells that are deemed important and crucial for the response are being considered in the new alternatives for lung toxicity testing and are being actively developed by several international projects (PATROLS for “Physiologically Anchored Tools for Realistic nanOmateriaL hazard aSsessment”) [149, 150].

#### Toxicogenomics as a useful tool for the identification and development of biomarkers for targeted testing of KEs

As alluded to earlier in the introduction, the wide scope of toxicogenomic data is optimal for defining the most robust biomarkers that are eligible for testing of AOP activation. Not only does the data provide deep insight into which genes are activated in relation to a specific AO, but it also provides the possibility to predict the activation of other biomolecules (transcription factors, proteins, non-coding RNAs), and to assess complex pathways and gene sets (signatures) correlated with a specific endpoint, such as an MIE, a KE or an AO, and in most cases, the signature can help assess multiple KEs at the same time. For example, in a study by Williams and Halappanavar [151], using datasets from public microarray repositories describing pulmonary diseases in mouse models and statistical methods such as bi-clustering and gene set enrichment analysis, essential features of altered lung transcriptomes following exposure to nanomaterials that are associated with lung-specific diseases were derived. Eight individual functionally related bi-clusters of genes showing similar expression profiles were identified [151] and were assigned to inflammation (chemokine activity), DNA binding, cell cycle, apoptosis, ROS and fibrosis processes. The genes provide robust sets of gene signatures that can be further validated to predict nanomaterial-induced lung responses. For examples, the gene sets assigned to inflammation and fibrosis bi-clusters in [151] were further pursued and a new predictive signature (PFS17) for the assessment of the KEs in AOP 173 predictive of lung fibrosis was developed [148]. In another study, using a large publicly available toxicogenomic database specifically for liver injury Kohonen et al.*,* defined [152, 153] a toxicogenomic space covering 1331 genes packed into 14 gene sets predictive of chemical-induced liver injury, including liver fibrosis. The identified biomarkers also relate to various toxicity pathways, including those shown to be induced by nanomaterials and in lung fibrosis, and are thus useful for bioinformatic modelling of molecular mechanisms related to nanotoxicity and other adverse outcomes. The selected gene sets and associated pathways describing the perturbed biology serve as a robust basis for quantifying transcriptomic signatures, probabilistic evaluation of MIE/KE activation, and prediction of the final AO [44, 152]. In addition, these multi-parametric biomarkers can be used for translating toxicogenomics data into parameters fit for use in conventional risk assessment routines, such as derivation of gene or pathway-based benchmark doses and points of departures [154, 155].

Lastly, owing to their comprehensiveness and sensitivity in differentiating subtle toxicity response induced by two individual nanomaterials of different properties [40, 42], high content toxicogenomic data can help define the structural properties of nanomaterials that are responsible for triggering an MIE and thus, an AO. For example, an approach that uses proteomic data to profile the protein corona of 84 gold nanoparticles as a basis for developing biological descriptors as input into quantitative structue activity relationship (Q) SAR modelling was recently described [156]. The integration of gene ontologies describing molecular function of the protein sets in the corona and using those to predict cellular uptake allowed the authors to draw conclusions on the molecular functions, i.e. the structure of proteins correlating with cellular uptake of nanoparticles. This approach can be extended to any type of omics data to be used as a basis for developing biological descriptors, including transcriptomic data, which was further implemented in a user-friendly interface referred to as the toxFlow tool [157].

These broad sets of biomarkers derived from toxicogenomics allows for the use of increasingly cheap high-throughput transcriptomic profiling methods to be used as first-tier hypothesis generators, e.g. with regard to which AOPs are most relevant to focus on from the network of AOPs. These steps are followed by more specific targeted methods coupled to rather advanced model systems serving as second-tier toxicity identifiers, as described previously by efforts detailing such tiered strategies [9, 44, 147, 158–160]. The application of omics in AOP-based risk assessment is a long-term goal, due to the need for standardized analysis pipelines, but in the meantime, omics can be used for identifying and describing MIEs and KEs, for development of quantitative AOPs, for AOP-linked read across, and as weight of evidence [48].

#### Challenges to implementing the AOP framework for nanomaterial research and risk assessment

As detailed in previous sections, AOPs potentially constitute next generation toxicology strategies and represent major paradigm shift in how toxicological research is conducted and interpreted, addressing the current and future chemical safety testing challenges. However, despite the availability of a well implemented and highly credible framework, extensive guidance for developing AOPs and a wide propaganda of their potential promises to reforming the existing risk assessment and regulatory decision making processes, there are significant challenges that have impeded their development and uptake [20]. The most crucial impediment in the context of nanomaterials is that there are no validated AOPs relevant to nanomaterial-induced AOs that can be used to support the decision making process. In general, the entire process, from the assembly of an AOP to its endorsement by the OECD WNT and WPHA, is very onerous and involves multiple steps: a) preparation and submission of an AOP proposal to the OECD EAGMST secretariat, b) review of the proposal by EAGMST, further consultation with WNT and WPHA for determining the regulatory relevance and susequent inclusion in the AOP work plan, and c) assembly of the full AOP in the AOP knowledgebase. The completely assembled AOP is then reviewed internally by the OECD EAGMST review committee for compliance with the AOP framework and externally by the OECD appointed subject matter experts for the accuracy of the biology presented [161]. Upon successful completion of these steps, the AOP is endorsed by the OECD WNT/WPHA and published on the OECD website [8]. Unless dedicated funds and sustainable human resources are available, this process can take more than a couple of years and in most cases, lack of sustainable resources force authors to abandon the efforts. The AOP knowledgebase lists more than 200 AOPs that are at different stages of development and less than 10 AOPs are fully assembled and endorsed [162]. Once endorsed, readiness of an AOP for a specific purpose (e.g., informing research gaps, regulatory decisions) has to be verified and will depend on the type of data used for building AOP modules (KEs, KERs) [8]. While putative AOPs can be used to inform data gaps, the quantitative AOPs can be used to support alternative testing strategies and generate data required for the chemical risk assessment [20]. A set of criteria have been proposed for assessing the readiness of the AOP for a given application and include confidence in the AOP assessed by the weight of evidence approach (confidence in KE essentiality, biological plausibility and empirical evidence), regulatory relevance of the KEs in the AOP (are the proposed KEs used for regulatory decision making) and the robustness and readiness of the bioassays proposed for KE measurement (standardized, validated, non-validated) [20]. In the regulatory context, the important impediment concerns the willingness of regulators to base a regulatory or a policy decision on AOP-informed testing strategies. To date, data generated from AOP-informed bioassays have not been used to make a regulatory decision. In the context of nanotoxicology, the concept of AOPs is new and the real challenges are extended to sheer availability of quality toxicology data to support the assembly of full AOPs. Significant efforts are made to develop and populate AOP knowledgebase with AOPs of relevance to nanomaterials; due to uncertainties in the available data and data gaps, at best, these AOPs can be viewed as putative or qualitative. Owing to the lack of mechanistic understanding, MIEs in nano relevant AOPs lack specificity. Moreover, insufficient understanding of how size-associated properties of nanomaterials impact the AOP transition from the MIE to an eventual AO, the domain of applicability cannot be specified. Thus, in the near term, the AOP framework can help researchers and decision makers in the nano community to identify KEs of regulatory relevance for the selection of targeted in vivo, in vitro or in silico assays, their design and development, the data generated from which can then be applied for prioritization of nanomaterials requiring further regulatory testing or to informing safe-by-design practices. The AOPs discussed in this review reflect only a few of the known pulmonary consequences of exposure to nanomaterials and thus, one route of exposure i.e., inhalation. At present, not all lung specific AOs potentially induced by nanomaterials are revealed and as more quality toxicity data becomes available, the list of AOPs describing inhalation toxicity of nanomaterials will expand. Parallel efforts are in place to construct AOPs of relevance to other routes of exposure to nanomaterials such as ingestion route. An additional emerging concern is that the nanotoxicology conducted in the last two decades has predominantly focused on simple first generation nanomaterials. The nanotechnology has evolved over this time period and a novel class of nanomaterials called ‘next generation nanomaterials’ or ‘advanced materials’, where one or more nanomaterials of different class and properties are complexed in a matrix, are introduced to the market and to the environment. Thus, the next important challenge for nano AOP enthusiasts is to determine how or can the existing AOPs relevant for the first generation nanomaterials be used to assess toxicity of next generation or advanced materials.

## Conclusions

Given the enormous burden placed on regulatory agencies to assess and manage an ever growing list of nanomaterials of various classes and properties, optimization and validation of alternative toxicity testing strategies including in vitro, ex vivo, and in silico methods, that aim to reduce or refine the involvement of animal testing methods, has become mandatory. However, in a regulatory context, to be effective, an alternative approach is expected to include a suite of tests that target different components of the underlying toxicity mechanism using different endpoints and assays. In the absence of in depth understanding of the involved biology and the biology being assessed by the alternatives, this exercise can rapidly reach a chaotic proportion and the initial objectives of rapid or timely and effective toxicity assessment can be easily lost. This review presents an overview of the AOP framework and its importance as support tool for effective development of alternative toxicity testing strategies for nanomaterials. Various AOPs of relevance to inhalation toxicity of nanomaterials that are currently under various stages of development are summarized and an AOP-informed tiered alternative testing strategy is proposed for the assessment of lung fibrosis. The review builds a solid case to 1) demonstrate how inclusion of AOP thinking that allows the design of mechanism-informed assays targeting most critical events in the path of a disease helps progress assessment of nanomaterials for their potential to induce human health impacts and 2) demonstrate how combination of high-content data (omics) to inform the development of an AOP and the design of AOP-informed assays targeting multiple key events, will expedite the progress in this field. Lastly, a few of the important issues that have plagued the development of AOPs in general and continue to impact their development as relevant to nanotoxicity are discussed.

## Data Availability

Not applicable.

## Abbreviations

AO: Adverse outcome
AOP: Adverse outcome pathway
BMD: Benchmark dose
CNT: Carbon nanotube
EAGMST: Extended Advisory Group on Molecular Screening and Toxicogenomics
ECM: Extracellular matrix
HARM: High aspect ratio material
HHRA: Human health risk assessment
KE: Key event
KER: Key event relationships
MIE: Molecular initiating event
MWCNT: Multi-walled carbon nanotube
OECD: Organisation for Economic Co-operation and Development
ROS: Reactive oxygen species
SAA: Serum Amyloid A
WPMN: Working Party for Manufactured Nanomaterials
